# Heterogeneity in Clinicopathological and PD-L1 Expression Profiles Across Mismatch Repair Protein Deficiency Patterns in Endometrial Carcinoma

**DOI:** 10.3390/cancers18182946

**Published:** 2026-09-11

**Authors:** Yunyun Xiao, Hao Yu, Danyu Huang, Shijia Liu, Yaping Wang, Ran Ren, Hanfu Hu, Shuoyan Wang, Haoyu Yu, Yi Li, Lu Han

**Affiliations:** 1 Department of Gynecology, Dalian Medical University, Dalian Maternal and Children’s Medical Group, Dalian 116044, China; 2 Department of Gynecology, Dalian University of Technology Affiliated Women and Children’s Hospital, Dalian 116033, China; 3 Department of Pathology, Dalian Maternal and Children’s Medical Group, Dalian University of Technology Affiliated Women and Children’s Hospital, Dalian 116033, China; 4 School of Future Technology, Dalian University of Technology, Dalian 116024, China; 5 School of Computer Science, Dalian University of Technology, Dalian 116024, China

**Keywords:** endometrial cancer, mismatch repair deficiency, PD-L1, immunotherapy, tumor heterogeneity

## Abstract

**Objectives**: This study aimed to investigate the relationships among mismatch repair (MMR) protein status, clinicopathological characteristics, and Programmed Death-Ligand 1 (PD-L1) expression in endometrial cancer, focusing specifically on heterogeneity within MMR-deficient (MMRd) tumors defined by distinct protein loss patterns. **Methods**: This retrospective study enrolled 675 patients with surgically confirmed endometrial cancer at a tertiary medical group between January 2017 and June 2025. Patients were stratified according to specific MMR protein loss patterns. Clinicopathological parameters, the International Federation of Gynecology and Obstetrics (FIGO) stage, and PD-L1 combined positive score were compared across groups. **Results**: The cohort comprised 502 MMR-proficient (MMRp) and 173 MMR-deficient (MMRd) patients. Based on their specific MMR protein loss patterns, the MMRd cases were further stratified into three subgroups: MutLαcomplex loss (MLH1 ± PMS2, *n* = 105), MutSαcomplex loss (MSH2 ± MSH6, *n* = 60), and combined MutSα/MutLαcomplex loss (*n* = 8). Compared with MMRp tumors, MMRd tumors exhibited significantly lower BMI, higher tumor grade, more frequent lymphovascular space invasion, and elevated PD-L1 expression. Moreover, the 2023 FIGO staging system demonstrated superior performance in capturing the locally aggressive features of MMRd tumors relative to the 2009 edition. Within the MMRd cohort, the overall difference in combined positive score reached nominal significance (*p* = 0.049) but did not survive FDR correction (q = 0.290). Nevertheless, at the 1% and 5% cutoffs, MutLα-deficient tumors consistently showed higher PD-L1 positivity than MutSα-deficient tumors, with both associations remaining significant after FDR adjustment (q = 0.046 for both). Further subgroup analysis within the MutLα loss pathway revealed that isolated MLH1 loss was associated with a lower rate of CPS ≥ 1% (85.7%) compared with isolated PMS2 loss (91.3%) and combined MLH1/PMS2 loss (89.3%), in contrast. No significant heterogeneity in PD-L1 expression was observed across the MutSα-deficient subgroups. **Conclusions**: MMRd endometrial cancer heterogeneity is primarily driven by MutSα and MutLα deficiency. MutSα loss associates with higher PD-L1 expression, suggesting a favorable immune profile warranting further study. Isolated MLH1 loss correlates with the lowest PD-L1 and aggressive features. These findings refine biological understanding and provide a hypothesis-generating rationale for biomarker-guided stratification in immunotherapy.

## Simple Summary

Analyzing 675 endometrial cancer patients, this study explored the relationship between mismatch repair (MMR) status, tumor features, and immunotherapy biomarkers. MMR-deficient (MMRd) tumors, comprising about 26% of cases, were more aggressive yet exhibited elevated PD-L1 expression. Importantly, MMRd tumors displayed significant heterogeneity: MutSα deficiency (MSH2/MSH6 loss) was associated with higher PD-L1 than MutLα deficiency (MLH1/PMS2 loss), whereas isolated MLH1 loss correlated with lower PD-L1 and a particularly aggressive course. The 2023 FIGO staging system proved superior to the 2009 edition in capturing the locally invasive features of MMRd tumors. Collectively, these findings underscore the clinical value of comprehensive testing for all four MMR proteins to enable refined subgrouping and guide personalized immunotherapy strategies.

## 1. Introduction

Endometrial carcinoma is the second most common malignancy of the female reproductive system, with its global incidence and mortality rates continuing to rise and showing a trend toward younger age at diagnosis [1,2]. According to the China Cancer Registry Annual Report, the crude incidence rate of endometrial cancer rose significantly from 6.20 per 100,000 in 2004 to 10.06 per 100,000 in 2017 (*p* < 0.001 for trend) [3]. Currently, the combination of immune checkpoint inhibitors and chemotherapy has become the first-line standard treatment for advanced and recurrent endometrial cancer. In second-line and subsequent therapies, immunotherapy (as monotherapy or in combination) is also prioritized for patients with molecular features such as mismatch repair deficiency/microsatellite instability-high (MMRd/MSI-H) or tumor mutational burden-high (TMB-H) [3,4,5]. These developments underscore a paradigm shift toward biomarker-driven precision immunotherapy in endometrial cancer management.

The mismatch repair (MMR) system maintains DNA replication fidelity through seven functional proteins, among which MLH1, MSH2, MSH6, and PMS2 are clinically most relevant [6]. These proteins operate as heterodimers: MSH2 with MSH6 forms the MutSα complex that recognizes base mismatches, while MLH1 with PMS2 constitutes the MutLα complex that initiates subsequent excision and resynthesis [7]. Published evidence has established that loss of MLH1/PMS2 is commonly linked to MLH1 promoter methylation, whereas loss of MSH2/MSH6 often accompanies germline mutations [8]. Approximately 70% of MMRd endometrial cancer cases are epigenetic (Epi-MMRd, methylated), and 30% are mutational (Mut-MMRd, unmethylated). A phase II trial (NCT02899793) of pembrolizumab in MMRd endometrial cancer reported a 100% response rate in Mut-MMRd patients (6/6) versus 44% in Epi-MMRd patients (8/18) [9]. Mut-MMRd patients also showed faster responses and significantly longer progression-free and overall survival, highlighting distinct immune responsiveness to PD-1 blockade between these molecular subtypes [9].

Currently, compared to PCR and NGS, immunohistochemistry (IHC) is recommended as the preferred detection method due to its lower cost, wide availability, and ability to simultaneously assess the loss of all four key MMR proteins [6,10]. Therefore, clarifying the relationship between the loss patterns of these four MMR proteins and potential immunotherapy response is crucial for guiding immune inhibitor drug selection and further elucidating the heterogeneity within MMRd tumors. PD-L1, a major ligand for the programmed death receptor-1 (PD-1), is a transmembrane glycoprotein belonging to the B7 co-stimulatory molecule family [11]. In normal tissues, PD-L1 is expressed on immune cells and some non-hematopoietic cells [12]. Its binding to PD-1 delivers an inhibitory signal, crucial for maintaining peripheral immune tolerance and preventing autoimmunity. In the tumor microenvironment, PD-L1 expression is aberrantly upregulated, driven either by oncogenic signaling or induced by cytokines like interferon-gamma released by tumor-infiltrating immune cells, making it a common biomarker for immune checkpoint inhibitors [13].

In MMRd endometrial cancer, PD-L1 expression levels are significantly higher than in MMR-proficient (MMRp) tumors, and are even higher in the Mut-MMRd subgroup than in the Epi-MMRd subgroup. This leads us to hypothesize that MSH2/MSH6 loss (primarily driven by germline mutations) may be associated with a stronger PD-L1-expressing phenotype compared to MLH1/PMS2 loss (often sporadic methylation). However, direct evidence for this hypothesis is lacking. Therefore, this study aims to systematically analyze the correlation between different MMR protein loss patterns and PD-L1 expression levels, as well as various clinicopathological parameters in endometrial cancer. This analysis seeks to provide an in-depth interpretation of the heterogeneity within MMRd tumors, offering a basis for more refined stratification for future immunotherapy.

## 2. Materials and Methods

### 2.1. Patient Selection

This retrospective study included patients diagnosed with endometrial carcinoma via postoperative pathology who underwent comprehensive staging surgery at Dalian Maternal and Children’s Medical Group from 1 January 2017, to 30 June 2025. Inclusion criteria: (1) Patients who underwent comprehensive staging surgery at our hospital and had histopathologically confirmed endometrial cancer; (2) Well-preserved pathological paraffin blocks or sections of sufficient quality for subsequent testing; (3) Age ≥ 18 years; (4) Complete clinical and pathological data (including surgical records, pathological reports, and follow-up information); (5) Informed consent provided by the patient or a legal guardian. Exclusion criteria: (1) Receipt of neoadjuvant therapy (chemotherapy, radiotherapy, endocrine therapy, targeted therapy, etc.) prior to surgery; (2) Pregnancy or lactation; (3) Concurrent malignancy in other organ systems, or a history of other malignancies within the past 5 years; (4) Severe cardiac, hepatic, or renal insufficiency, or uncontrolled systemic infection; (5) Inadequate tissue samples for IHC evaluation, including missing paraffin blocks, insufficient tumor content, or uninterpretable internal controls.

**Figure 1:**
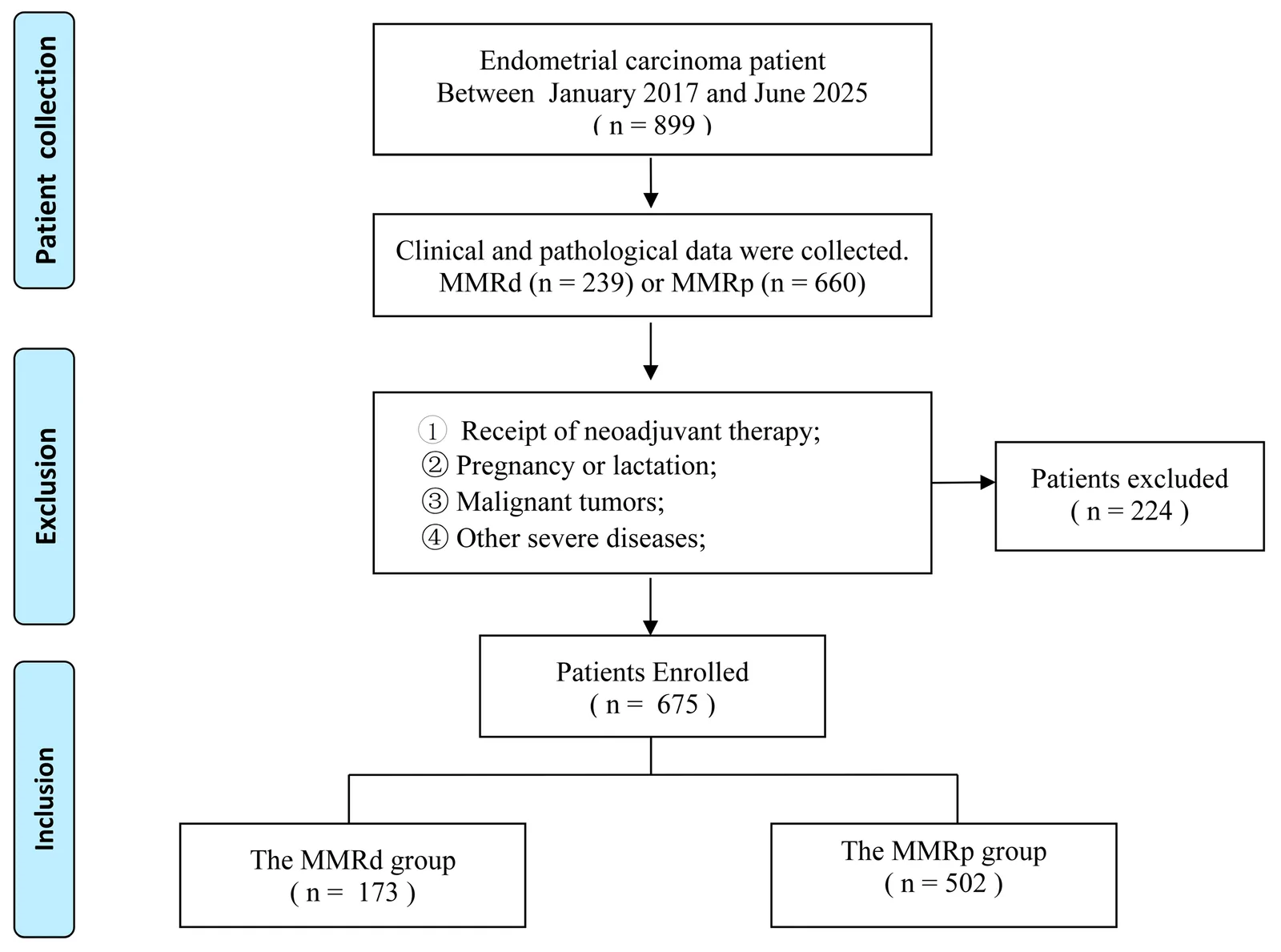
Workflow of the research.

Clinical and pathological data were collected, encompassing basic patient information, clinical diagnosis and medical history, surgical approach, and detailed tumor-related parameters—specifically tumor size, histological type, differentiation grade, depth of myometrial invasion, lymphovascular invasion status, and lymph node metastasis. Estrogen receptor (ER) and progesterone receptor (PR) expression data were also included. The workflow of this study is presented in Figure 1. The study protocol was approved by the Ethics Committee of Dalian Maternal and Children’s Medical Group (FEJT-KXSC-2025-21).

### 2.2. Section Staining and Scoring

Formalin-fixed, paraffin-embedded (FFPE) tumor tissue blocks were collected from all enrolled patients and serially sectioned into 4-μm-thick slices, with five consecutive sections prepared per case for subsequent immunohistochemical (IHC) staining. IHC was performed using primary antibodies against MLH1 (clone MAB-0789, MXB Biotechnologies, Fuzhou, China), MSH2 (clone MAB-0836, MXB Biotechnologies, Fuzhou, China), MSH6 (clone ZA-0541, ZSGB-Bio Co., Ltd., Beijing, China), PMS2 (clone ZA-0542, ZSGB-Bio Co., Ltd., Beijing, China), and PD-L1 (clone E1L3N, Cell Signaling Technology, Danvers, MA, USA). Appropriate positive and negative controls were included in each staining run to ensure assay validity. All stained slides were independently evaluated by two experienced pathologists (each with over 20 years of practice) who were blinded to all clinical data; any interpretive discrepancies were resolved by consensus review.

MMR protein expression was assessed primarily by evaluating nuclear staining intensity and distribution in tumor cells, with adjacent non-neoplastic cells (including stromal cells, lymphocytes, and normal endometrial glands) serving as internal positive controls. A case was classified as MMR-deficient (MMRd) if complete loss of nuclear expression was observed for any of the four MMR proteins (MLH1, PMS2, MSH2, or MSH6), provided that internal controls showed adequate staining. Conversely, MMR-proficient (MMRp) status was assigned when all four proteins exhibited retained nuclear expression. Cases with equivocal staining, weak internal controls, or uninterpretable staining patterns were subjected to repeat IHC on additional sections and were excluded if findings remained inconclusive.

PD-L1 expression was evaluated by IHC on FFPE tissue sections using the rabbit monoclonal anti-PD-L1 antibody clone E1L3N. IHC staining was performed on a fully automated immunostainer (Ventana BenchMark ULTRA, Roche Diagnostics, Tucson, AZ, USA) using the OptiView DAB IHC Detection Kit (Ventana Medical Systems, Inc., Tucson, AZ, USA). Antigen retrieval was carried out with CC1 standard buffer (pH 8.5) for 64 min at 100 °C, followed by incubation with the primary antibody at a dilution of 1:200 for 32 min at 37 °C. Positive and negative controls were included in each run: placental tissue served as the positive control, and omission of the primary antibody served as the negative control. Internal positive controls (normal endometrial epithelial and stromal cells) were also evaluated to confirm tissue integrity and staining adequacy; cases with uninterpretable internal controls were excluded. Images of immunohistochemically stained tissue sections were shown in Supplementary Figure S1.

PD-L1 expression was assessed using the Combined Positive Score (CPS), defined as the number of PD-L1-staining cells (tumor cells, lymphocytes, and macrophages) divided by the total number of viable tumor cells, multiplied by 100. CPS was calculated as follows:

$$CPS=\frac{Number\;of\;PD-L1-positive\;cells(tumor\;cells+lymphocytes+macrophages)}{Total\;number\;of\;viable\;tumor\;cells}\times 100$$

PD-L1 positivity was defined as any partial or complete membranous staining of any intensity on viable tumor cells and mononuclear inflammatory cells (lymphocytes and macrophages), in accordance with the Dako 22C3 pharmDx CPS scoring criteria. The CPS was scored by two independent pathologists blinded to clinical data; discrepancies were resolved by consensus review. For descriptive purposes, CPS values were categorized into five tiers: CPS < 1%, 1% ≤ CPS < 5%, 5% ≤ CPS < 10%, 10% ≤ CPS < 50%, and CPS ≥ 50%. In addition, to facilitate comparability with published literature and clinical trial benchmarks, we also reported PD-L1 positivity using the conventional CPS cutoffs of ≥1% and ≥5% in MutLα and MutSα loss subgroups.

Estrogen receptor (ER) and progesterone receptor (PR) expression were evaluated using the standardized Allred scoring system. The assessment was conducted by first examining tissue core integrity to ensure analytical suitability, followed by the evaluation of the proportion of positively stained tumor cells and the staining intensity. The proportion score was assigned as follows: 0 for no staining, 1 for <10%, 2 for 10–25%, 3 for 26–50%, 4 for 51–75%, and 5 for 76–100% positive cells. The intensity score was graded as: 0 for none, 1 for weak, 2 for moderate, and 3 for strong staining. The total Allred score (ranging from 0 to 8) was calculated by summing the proportion and intensity scores, with a higher total score indicating stronger receptor expression.

### 2.3. Statistical Analysis

Patients were stratified into MMRd and MMRp groups according to MMR protein expression status, with further subgroup analyses based on distinct MMR loss patterns to compare clinicopathological characteristics, PD-L1, ER, and PR expression. All statistical analyses were performed using SPSS version 26.0 (IBM Corp., Armonk, NY, USA).

For continuous variables, normality was assessed using the Shapiro–Wilk test, and homogeneity of variances was assessed using Levene’s test. Normally distributed data with equal variances were expressed as mean ± standard deviation and compared using Student’s *t*-test. When normality was satisfied but variances were unequal, Welch’s *t*-test was applied. For non-normally distributed data, results were expressed as median (interquartile range) and compared using the Mann–Whitney U test.

Categorical variables were summarized as frequencies and percentages. Group comparisons were performed using the Pearson chi-square test when all expected frequencies were >5. Yates’ continuity correction was applied when expected frequencies ranged from 1 to 5, and Fisher’s exact test was used when any expected frequency was <1 or the total sample size was <40. For ordinal categorical variables, the Mantel-Haenszel test for trend was additionally applied where appropriate. To avoid unreliable statistical inference, if the sample size of either group was <10, inferential testing was omitted, and variables were reported descriptively (frequencies and percentages) without *p*-values.

Effect sizes were calculated for key comparisons: Cohen’s d for continuous variables, Cramer’s V for categorical associations, and odds ratios (OR) with 95% confidence intervals where applicable. Given that multiple hypothesis tests were performed across several tabular comparisons, a two-sided *p*-value < 0.05 was considered statistically significant only when adjusted for multiplicity. Specifically, multiplicity-adjusted *p*-values (false discovery rate, FDR-q) using the Benjamini–Hochberg method were applied across all comparisons to control the Type I error rate, and statistical significance was determined based on FDR-q < 0.05.

## 3. Results

### 3.1. MMRd Versus MMRp: Clinicopathological and PD-L1 Profiles

**Figure 2:**
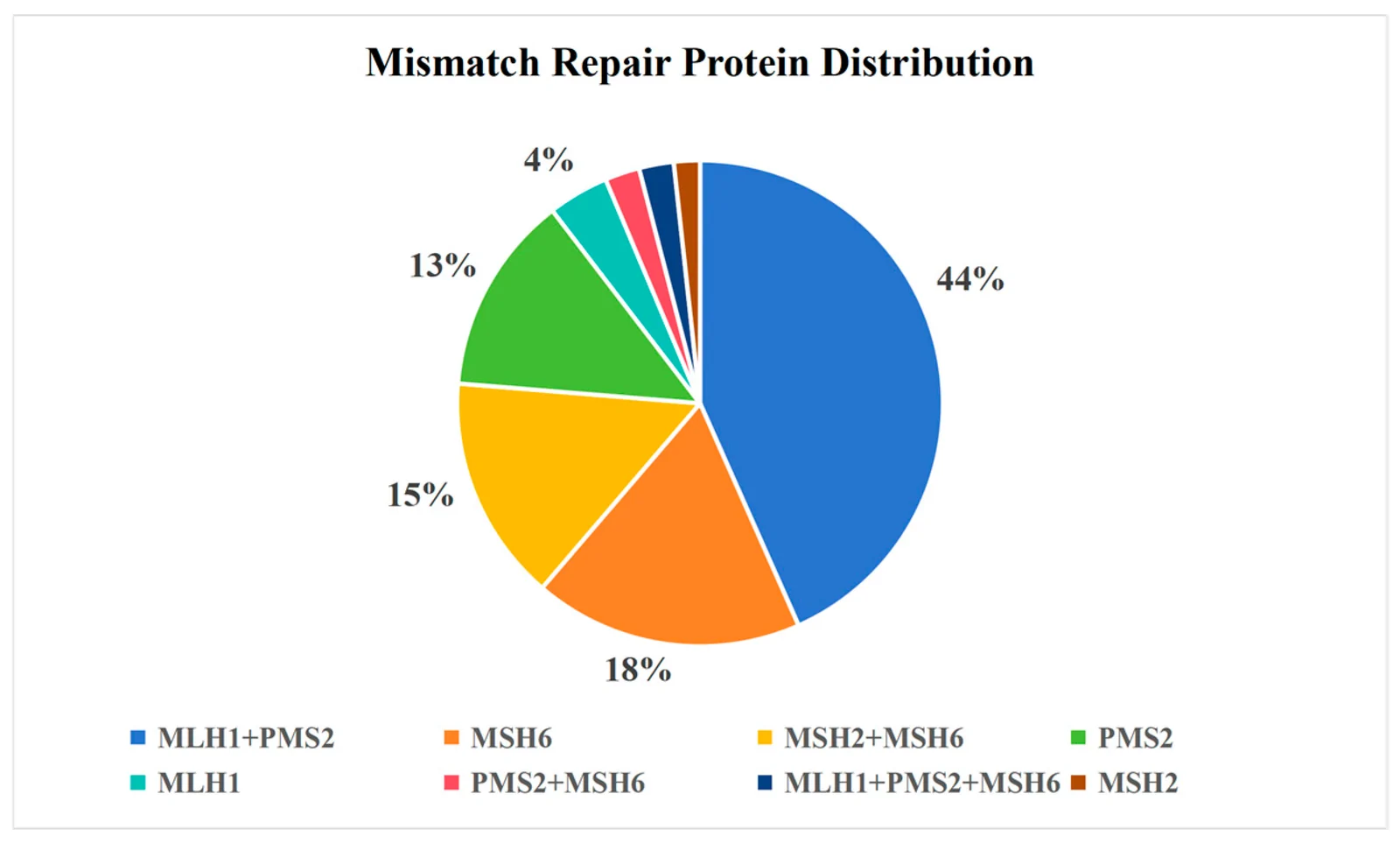
Detailed mismatch repair protein loss patterns.

This study included 675 patients with endometrial cancer, of whom 173 (25.6%) were classified as MMRd and 502 (74.4%) as MMRp (Table 1). Detailed mismatch repair protein loss patterns were shown in Figure 2. Patients in the MMRd group had a significantly lower body mass index (BMI) than those in the MMRp group (median 24.74 vs. 25.72, *p* = 0.015, FDR-q = 0.034). Due to the non-parametric distribution, Cohen’s d is not applicable; the corresponding effect size r was negligible (<0.1), indicating minimal clinical relevance. Pathologically, MMRd tumors displayed more aggressive features, including a higher proportion of grade 3 histology (24.3% vs. 12.9%, *p* = 0.002, FDR-q = 0.009, Cramer’s V = 0.136), a more aggressive phenotype (24.3% vs. 15.2%, *p* = 0.006, FDR-q = 0.025, Cramer’s V = 0.101), and more frequent extensive lymphovascular space invasion (13.3% vs. 6.8%, *p* = 0.010, FDR-q = 0.031, Cramer’s V = 0.117). Regarding tumor staging, no significant difference was observed between MMRd and MMRp tumors using the 2009 FIGO system (*p* = 0.594, FDR-q = 0.711). However, when applying the 2023 FIGO staging system, a statistically significant difference emerged (*p* = 0.016, FDR-q = 0.034), with MMRd tumors showing a higher proportion of stage IIC disease (19.1% vs. 10.8%) and a lower frequency of stage I tumors (57.8% vs. 67.9%) compared with MMRp tumors. No significant differences were observed in age, lymph node metastasis, cervical involvement, or hormone receptor scores between the two groups. ER and PR scores showed no significant differences between MMRd and MMRp groups (both median 6), with *p*-values of 0.621 and 0.102, and FDR-q of 0.711 and 0.175, respectively.

PD-L1 expression, evaluated by the combined positive score (CPS), differed significantly between the MMRd and MMRp groups (*p* < 0.001, FDR-q < 0.001, Cramer’s V = 0.381). Compared with MMRp tumors, MMRd tumors showed lower frequencies of CPS-negative (<1%) and CPS-low (1–5%) cases (6.4% vs. 16.5% and 31.2% vs. 40.5%, respectively), and higher proportions of CPS-moderate-high (10–50%) and CPS-high (≥50%) cases (26.6% vs. 14.8% and 15.0% vs. 0.9%, respectively). This distribution shift reflects a relatively higher PD-L1 expression profile in MMRd tumors than in their MMRp counterparts.

Multivariate logistic regression (including BMI, tumor grade, FIGO 2023 stage, and PD-L1 CPS) identified three independent predictors of MMR status, but not stage. Multivariate logistic regression showed that higher BMI was associated with decreased odds of MMRd (aOR = 0.948, 95% CI: 0.903–0.992, *p* = 0.024), while higher histologic grade and higher PD-L1 CPS were associated with increased odds of MMRd (aOR = 1.366, 95% CI: 1.042–1.795, *p* = 0.025; and aOR = 1.229, 95% CI: 1.080–1.397, *p* = 0.002, respectively). FIGO 2023 stage was not a significant predictor (aOR = 0.999, 95% CI: 0.866–1.147, *p* = 0.992). These findings indicate that BMI, grade, and PD-L1 expression independently influence MMR status.

### 3.2. MutSα Versus MutLα Deficiency: Clinicopathological and PD-L1 Profiles

The 173 MMRd endometrial cancer patients were classified into three subgroups based on their specific mismatch repair protein loss patterns: MutSα complex loss (MSH2 ± MSH6, *n* = 60), MutLα complex loss (MLH1 ± PMS2, *n* = 105), and combined MutSα/MutLα complex loss (*n* = 8, comprising 4 cases of PMS2 + MSH6 co-loss and 4 cases of MLH1 + PMS2 + MSH6 loss) (Supplementary Table S1); detailed loss patterns are depicted in Figure 2. Given the limited number of cases (*n* = 8) in the combined-loss subgroup, we restricted our comparative analyses to the MutSα-loss and MutLα-loss groups to ensure statistical robustness.

No significant intergroup differences were observed for age, BMI, depth of myometrial invasion, lymph node metastasis, or FIGO stage 2009. However, the MutSα loss subgroup exhibited a higher proportion of grade 3 tumors (35.0% vs. 19.0%, *p* = 0.067, FDR-q = 0.290, Cramer’s V = 0.181, Table 2), though not statistically significant. While the overall histological type distribution was nominally significant (*p* = 0.019), the effect was not robust to adjustment for multiple comparisons (FDR q = 0.245, Cramer’s V = 0.246). The trend appeared largely driven by clear cell carcinoma, which was observed solely in the MutSα-deficient subgroup (5/60). This finding raises the possibility of a subtype-specific phenotype, though it clearly requires validation in larger, independent study populations. ER and PR scores were comparable between MutLα and MutSα subgroups, with *p*-values of 0.808 and 0.405, and FDR-q of 0.885 and 0.550, respectively.

PD-L1 expression, assessed by the CPS, showed a nominally significant difference between the two MMR-deficient subgroups (MutLα-deficient vs. MutSα-deficient, *p* = 0.049, Cramer’s V = 0.240). However, this association did not retain significance after correction for multiple testing (FDR-q = 0.290). Furthermore, PD-L1 positivity, defined at two CPS cutoffs (1% and 5%), was significantly associated with MMR deficiency status, as shown in Supplementary Table S2. At the 1% cutoff, the positivity rate was 89.5% (94/105) in the MutLα-deficient subgroup versus 100% (60/60) in the MutSα-deficient subgroup (*p* = 0.023, Cramer’s V = 0.177). At the 5% cutoff, positivity was 54.3% (57/105) vs. 71.7% (43/60), respectively (*p* = 0.028, Cramer’s V = 0.171). Both associations remained statistically significant after false discovery rate correction (FDR-q = 0.046 for both), indicating that the observed differences are unlikely to be false positives despite multiple testing.

Multivariate logistic regression, adjusted for tumor grade, FIGO 2023 stage, and PD-L1 CPS, identified CPS as the only independent predictor of MutSα versus MutLα deficiency. Higher CPS significantly increased the odds of MutSα deficiency (aOR = 1.418, 95% CI: 1.082–1.877, *p* = 0.013). In contrast, neither grade (aOR = 1.400, *p* = 0.151) nor stage (aOR = 1.012, *p* = 0.926) showed a significant association. Thus, PD-L1 expression may serve as a distinguishing feature between these two MMRd subgroups.

### 3.3. Heterogeneity Within MutLα Deficiency Subgroups: Clinicopathological and PD-L1 Profiles

Among the 105 cases with MutLα complex loss, three distinct subgroups were identified based on specific loss patterns: combined MLH1/PMS2 loss (*n* = 75), isolated PMS2 loss (*n* = 23), and isolated MLH1 loss (*n* = 7) (Table 3). Age, grade, and stage appeared comparable across these groups, although the isolated MLH1 loss subgroup exhibited a higher median BMI (29.30) than the combined loss (24.77) and isolated PMS2 loss (25.30) subgroups. Regarding histological subtype distribution, endometrioid carcinoma predominated in all three groups; however, the combined loss group additionally included mixed and mucinous carcinomas, isolated PMS2 loss harbored one carcinosarcoma, and isolated MLH1 loss featured one neuroendocrine carcinoma. These observations suggest that divergent differentiation may be associated with specific loss patterns. ER and PR scores were largely similar across the three MutLα subgroups, with median ER ranging from 5.5 to 6.5 and PR from 4.5 to 6.5.

With respect to PD-L1 expression assessed by CPS, the frequency of CPS-negative (<1%) and CPS-low (1–5%) cases was broadly similar across the three subgroups, with isolated MLH1 loss showing the highest proportions (14.3% and 57.1%, respectively), while no marked intergroup differences were observed in the moderate range (5–10%). In the higher expression categories, only the combined loss group exhibited CPS ≥ 50% (17.3%) and additionally had 18.7% of cases in the 10–50% range. In contrast, the isolated PMS2 loss group was predominantly enriched in the 10–50% category (47.8%) and had no cases with CPS ≥ 50%.

### 3.4. Heterogeneity Within MutSα Deficiency Subgroups: Clinicopathological and PD-L1 Profiles

Among the 60 patients with MutSα complex loss, three distinct protein loss patterns were identified: combined MSH2/MSH6 loss (*n* = 26), isolated MSH6 loss (*n* = 31), and isolated MSH2 loss (*n* = 3) (Table 4). While clinicopathological features such as BMI, histology, grade, and local invasion appeared comparable across the subgroups, age at diagnosis showed a notable gradient: patients with isolated MSH6 loss had the oldest mean age (58.7 years), whereas those with isolated MSH2 loss were the youngest (mean 45.0 years), hinting at potential differences in underlying germline susceptibility or tumor biology. Regarding FIGO 2023 stage distribution, the combined MSH2/MSH6 loss group exhibited the highest frequency of stage IIC disease (38.5%), while isolated MSH6 loss predominantly presented with stage IA2 tumors (41.9%), and isolated MSH2 loss showed a relatively elevated proportion of stage III disease (33.3%). ER and PR scores were comparable across the three MutSα subgroups, with mean ER ranging from 5.27 to 5.71 and PR from 3.83 to 5.07.

With respect to PD-L1 expression assessed by CPS, the intergroup distributions were broadly similar. Notably, all cases were CPS-positive (≥1%), and high expression (CPS ≥ 50%) was consistently observed across subgroups, with rates of 23.1% in the combined loss group, 33.3% in isolated MSH2 loss, and 19.4% in isolated MSH6 loss.

## 4. Discussion

Endometrial cancer has the highest frequency of MMRd tumors among gynecologic malignancies, accounting for approximately 30% of cases [14]. These tumors exhibit a high mutational burden and neoantigen load, fostering an immunologically active microenvironment with prominent TIL infiltration and upregulated PD-L1 [15]. This immune activation drives exceptional responsiveness to PD-1 blockade, with ORRs of 48–57% in advanced disease versus only 3–20% in MMRp tumors [16,17]. The addition of PD-(L)1 inhibitors to chemotherapy further improves survival in advanced MMRd disease. However, MMRd tumors are not homogeneous; differences in the underlying mechanism of MMR deficiency (epigenetic versus germline/somatic mutations) and in specific protein loss patterns (MutLα vs. MutSα) are associated with distinct clinicopathological features and immune profiles [9,18]. Therefore, substratification of MMRd tumors by their specific MMR loss patterns is essential to guide personalized immunotherapy and optimize clinical outcomes.

This study analyzed 675 endometrial cancer patients, comprising 173 MMRd and 502 MMRp cases, and revealed that MMRd tumors were associated with lower BMI, higher tumor grade and more frequent lymphovascular space invasion. In keeping with previous reports, MMRp tumors exhibited a higher BMI than MMRd tumors, a difference likely reflecting divergent tumorigenic pathways. While obesity, a well-established risk factor for endometrial cancer, appears to contribute more to MMRp tumor development. While no significant difference in stage distribution was observed between the two groups under the FIGO 2009 system, the FIGO 2023 staging criteria yielded a difference that approached statistical significance (*p* = 0.016), largely driven by stage IIC, which accounted for 19.1% of MMRd cases versus 10.8% of MMRp cases. We consider that the FIGO 2023 staging system, particularly its newly defined stage IIC, captures these aggressive features more accurately than the 2009 edition. It is worth noting that some studies have questioned the discriminatory ability among FIGO 2023 stage II substages, suggesting that stage IIC may represent a heterogeneous cohort [19,20]. Additionally, PD-L1 expression was markedly elevated in MMRd tumors, especially with a higher proportion of cases exhibiting moderate-to-high combined positive scores. Notably, 15.0% of MMRd tumors achieved a CPS ≥50%, compared with only 0.9% in the MMRp group. This finding aligns with a large-scale analysis of 529 endometrial carcinoma cases, which reported that PD-L1 and LAG-3 expressions in the MMRd and p53-mutant groups were significantly higher than in the MMRp group (*p* < 0.001) [21]. A systematic review further demonstrated that PD-L1 is more frequently expressed in MSI tumors (49%) than in microsatellite-stable tumors (33.5%), and PD-L1 expression varies across molecular subtypes, being highest in POLE-mutated tumors (76.56%) and serous carcinomas (73%) [22]. Clinically, the addition of PD-(L)1 inhibitors to platinum-based chemotherapy has been shown to significantly improve both overall survival (HR 0.36, 95% CI 0.21 to 0.62) and progression-free survival (HR 0.35, 95% CI 0.23 to 0.53) in patients with advanced or recurrent MMRd endometrial cancer [23]. These multidimensional findings collectively support MMRd as a key predictive biomarker for immunotherapy response in endometrial cancer.

Among the 173 MMRd patients, substantial heterogeneity emerged according to specific MMR protein loss patterns. The MutLα deficiency subtype (MLH1 ± PMS2 loss, 60.7%) constituted the largest group and was predominantly associated with endometrioid carcinoma. In contrast, the MutSα deficiency subtype (MSH2 ± MSH6 loss, 34.7%) encompassed all clear cell carcinomas and exhibited higher tumor grade features. We acknowledge that de Freitas et al. reported MSH2/MSH6 loss with less aggressive features, whereas our cohort shows MutSα loss enriched for grade 3 tumours and harbouring all clear cell carcinomas. These discrepancies may reflect differences in cohort composition, ethnicity, stage distribution, or small subgroup sizes [24]. Rare co-loss patterns, namely PMS2 + MSH6 and MLH1 + PMS2 + MSH6, were also identified, each accounting for 2.3% of MMRd cases.

In addition to the differences observed at the 1% and 5% thresholds, the MutSα-loss subgroup displayed an overall elevation in PD-L1 expression levels, with no tumors falling into the CPS-negative category (<1%) and a notably higher frequency of high-level expression (CPS ≥ 50%) relative to the MutLα-loss subgroup (21.7% vs. 12.4%, respectively). Within the MutLα-loss pathway, substantial heterogeneity was observed across its three molecular subgroups. Combined MLH1/PMS2 loss showed a notable potential for high PD-L1 expression, with 17.3% of cases reaching CPS ≥ 50%. Isolated PMS2 loss was predominantly clustered in the moderate-to-high range (47.8% with CPS 10–50%), whereas isolated MLH1 loss was uniformly PD-L1-low, with all cases falling below CPS < 10%, and was uniquely associated with neuroendocrine carcinoma. In the MutSα-loss pathway, combined MSH2/MSH6 loss exhibited the highest frequency of stage IIC disease (38.5%) and the greatest proportion of grade 3 tumors, suggesting a particularly aggressive clinical behavior.

The MMR system safeguards genomic integrity by correcting mismatches and insertion/deletion loops generated during DNA replication, a process coordinated by two heterodimeric complexes: MutSα (MSH2 and MSH6) for error recognition and MutLα (MLH1 and PMS2) for excision and resynthesis. The underlying mechanisms of MMR deficiency differ fundamentally between these two pathways. Loss of MutLα is most commonly attributable to somatic MLH1 promoter hypermethylation, which accounts for approximately 70–75% of MMRd endometrial cancers in published series [25]. Conversely, loss of MutSα is more frequently associated with germline mutations in the corresponding MMR genes, indicating a higher likelihood of Lynch syndrome [26,27]. However, we acknowledge that these mechanistic attributions are derived from the literature and were not directly tested in our cohort, as detailed in the Limitations section. This mechanistic dichotomy has important clinical implications, as Lynch syndrome-associated MMRd tumors have been reported to display significantly higher CD3^+^, CD8^+^, and PD-1^+^ T-cell infiltration compared with sporadic MLH1-methylated cases, and patients with Lynch syndrome-associated endometrial cancers show a trend toward better recurrence-free survival [28,29].

These findings align with and extend prior observations. A study by Freitas et al. reported that relative to MLH1/PMS2 loss, MSH2/MSH6 loss in MMRd tumors was associated with earlier diagnosis, smaller tumor size, and less myometrial invasion, LVSI, and lymph node metastasis [24]. Consistent with our observations, patients with MutSα deficiency might exhibit better immune status, prognosis, and immunotherapy benefits than those with MutLα deficiency. Conversely, MLH1 loss has been associated with worse outcomes and lower CD8^+^ tumor-infiltrating lymphocyte densities [28,29,30,31]. A large cohort study of 899 MMRd endometrial cancers further demonstrated that patients with MLH1 loss (80% of the cohort) were older with higher BMIs and had more deeply invasive tumors and more lymph node metastases, with 18% recurring and 10% dying of disease compared with 7% and 4% among those with MSH2/MSH6 or isolated PMS2 loss, respectively [30,31].

This study yields three clinically actionable insights. First, a comprehensive assessment of all four MMR proteins is indispensable, as specific loss patterns enable the discrimination of molecular subtypes with distinct biological behavior and immune phenotypes. Second, therapeutic strategies should be calibrated to the inherent heterogeneity of MMRd tumors: patients with MSH2/MSH6 loss, characterized by uniformly elevated PD-L1 expression, may represent optimal candidates for single-agent PD-1/PD-L1 blockade, whereas those with MLH1/PMS2 loss—particularly cases with low PD-L1 expression—may require upfront combination approaches to overcome potential primary resistance. Third, future clinical trials should incorporate prospective stratification by specific MMR protein loss patterns to precisely evaluate the differential efficacy of immunotherapeutic regimens, thereby advancing endometrial cancer management toward true precision medicine.

Several limitations of this study should be acknowledged. First, the absence of systematic germline testing and MLH1 promoter methylation analysis precluded definitive determination of the molecular basis of MMR deficiency, especially in the MutSα-deficient subgroup. Consequently, we cannot attribute the observed clinicopathological or PD-L1 differences specifically to epigenetic versus mutational drivers, and further molecular characterization is clearly warranted. Second, the small number of cases in rare subgroups (including isolated MLH1 loss, isolated MSH2 loss, and the combined PMS2 + MSH6 and MLH1 + PMS2 + MSH6 loss patterns) limited statistical power, preventing robust conclusions on their clinical and PD-L1 profiles; this underscores the need for larger collaborative investigations. Third, as a retrospective single-center analysis with a modest sample size, our findings require validation in prospective multicenter studies with larger, more diverse cohorts.

## 5. Conclusions

This systematic analysis of 675 endometrial cancer patients in a Chinese cohort confirms that MMRd tumors are characterized by both aggressive clinicopathological features and enhanced PD-L1 expression. The 2023 FIGO staging system demonstrated superior accuracy in capturing their locally invasive features compared with the 2009 edition. Significant heterogeneity within MMRd tumors was delineated by specific protein loss patterns; MutSα deficiency was associated with higher PD-L1 expression than MutLα deficiency, whereas the latter displayed marked diversity in PD-L1 levels, with isolated MLH1 loss linked to the lowest expression. No significant heterogeneity was observed within the MutSα deficiency subgroup. Collectively, these findings highlight the clinical utility of comprehensive immunohistochemical testing for all four MMR proteins to identify distinct loss patterns and heterogeneous subgroups. This refined stratification reinforces MMRd as a predictive biomarker for immunotherapy and improves diagnostic accuracy, prognostic assessment, and personalized treatment selection.

## Figures and Tables

**Table 1:** Clinicopathological characteristics of patients with mismatch repair deficient (MMRd) versus mismatch repair proficient (MMRp) endometrial cancer.

Characteristics	MMRd (*n* = 173)	MMRp (*n* = 502)	*p*-Value	FDR-q
Age, median (IQR)	55 (51, 61)	56 (51, 63)	0.132	0.218
BMI, median (IQR)	24.7 (23.0, 27.9)	25.7 (23.5, 28.6)	0.015 *	0.034 *
Pathology			0.067	0.055
endometriod	154 (89%)	455 (90.6%)		
carcinosarcoma	1 (0.6%)	6 (1.2%)		
mixed	8 (4.7%)	12 (2.4%)		
serous	0 (0%)	14 (2.8%)		
clear cell	5 (2.9%)	8 (1.6%)		
other	5 (2.9%)	7 (1.4%)		
Differentiation			0.002 *	0.009 *
G1	107 (61.8%)	364 (72.5%)		
G2	24 (13.9%)	73 (14.5%)		
G3	42 (24.3%)	65 (12.9%)		
Invasive			0.006 *	0.025 *
absent	131 (75.7%)	425 (84.8%)		
present	42 (24.3%)	76 (15.2%)		
Myometrial invasion			0.215	0.299
absent	7 (4%)	40 (8%)		
<1/2	123 (71.1%)	340 (67.7%)		
≥1/2	43 (24.9%)	122 (24.3%)		
LVSI			0.010 *	0.031 *
absent	103 (59.5%)	350 (69.7%)		
local	47 (27.2%)	118 (23.5%)		
extensive	23 (13.3%)	34 (6.8%)		
Pelvic LNM			0.678	0.711
absent	156 (90.2%)	447 (89%)		
present	17 (9.8%)	55 (11%)		
Aortic LNM			0.858	0.858
absent	167 (96.5%)	486 (96.8%)		
present	6 (3.5%)	16 (3.2%)		
Cervical invasion			0.180	0.277
absent	149 (86%)	451 (89.8%)		
present	24 (14%)	51 (10.2%)		
Stage 2009			0.594	0.711
IA	111 (64.2%)	338 (67.3%)		
IB	28 (16.2%)	70 (13.9%)		
II	15 (8.7%)	27 (5.4%)		
IIIA	2 (1.2%)	10 (2%)		
IIIB	0 (0%)	2 (0.4%)		
IIIC1	11 (6.4%)	40 (8%)		
IIIC2	6 (3.5%)	15 (3%)		
Stage 2023			0.016 *	0.034 *
I	100 (57.8%)	341 (67.9%)		
IIA	8 (4.6%)	21 (4.2%)		
IIB	13 (7.5%)	21 (4.2%)		
IIC	33 (19.1%)	54 (10.8%)		
III	19 (11%)	65 (12.9%)		
ER score, median (IQR)	6 (5, 7)	6 (6, 7)	0.621	0.711
PR score, median (IQR)	6 (4, 7)	6 (5.5, 7)	0.102	0.175
CPS, *n* (%)			<0.001 *	<0.001 *
<1%	11 (6.4%)	75 (16.5%)		
1% ≤ CPS < 5%	54 (31.2%)	184 (40.5%)		
5% ≤ CPS < 10%	36 (20.8%)	124 (27.3%)		
10% ≤ CPS < 50%	46 (26.6%)	67 (14.8%)		
≥50%	26 (15%)	4 (0.9%)		
NR	0	48 (9.6%)		

Abbreviations: MMRd, mismatch repair deficient; MMRp, mismatch repair proficient; IQR, interquartile range; BMI, body mass index; LVSI, lymphovascular space invasion; LNM, lymph node metastasis; ER, estrogen receptor; PR, progesterone receptor; CPS, combined positive score; *, <0.05.

**Table 2:** Clinicopathological characteristics of MMRd endometrial cancer patients stratified by specific protein loss patterns.

Characteristics	MutLα	MutSα	*p*-Value	FDR-q
	(*n* = 105)	(*n* = 60)		
Age, mean ± sd	55.5 ± 8.5	55.3 ± 8.5	0.868	0.999
BMI, median (IQR)	25 (23.4, 28.6)	24.3 (23.0, 27.3)	0.161	0.371
Pathology			0.019 *	0.245
endometriod	98(93.3%)	49 (81.7%)		
clear cell	0 (0%)	5 (8.3%)		
mixed	4 (3.8%)	4 (6.7%)		
other	3 (2.9%)	2 (3.3%)		
Differentiation			0.067	0.290
G1	69 (65.7%)	33 (55%)		
G2	16 (15.2%)	6 (10%)		
G3	20 (19%)	21 (35%)		
Invasive			0.126	0.371
absent	82 (78.8%)	41 (68.3%)		
present	22 (21.2%)	19 (31.7%)		
Myometrial invasion			0.425	0.642
absent	3 (2.9%)	3 (5%)		
<1/2	78 (74.3%)	39 (65%)		
≥1/2	24 (22.9%)	18 (30%)		
LVSI			0.284	0.528
absent	60 (57.1%)	37 (61.7%)		
local	27 (25.7%)	18 (30%)		
extensive	18 (17.1%)	5 (8.3%)		
Pelvic LNM			0.923	0.999
absent	94 (89.5%)	54 (90%)		
present	11 (10.5%)	6 (10%)		
Aortic LNM			1.000	0.999
absent	101 (96.2%)	58 (96.7%)		
present	4 (3.8%)	2 (3.3%)		
Cervical invasion			0.445	0.642
absent	92 (87.6%)	50 (83.3%)		
present	13 (12.4%)	10 (16.7%)		
Stage 2009			0.491	0.692
IA	70 (66.7%)	35 (58.3%)		
IB	16 (15.2%)	11 (18.3%)		
II	6 (5.7%)	8 (13.3%)		
IIIA	2 (1.9%)	0 (0%)		
IIIC1	7 (6.7%)	4 (6.7%)		
IIIC2	4 (3.8%)	2 (3.3%)		
Stage 2023			0.049 *	0.290
IA1	3 (2.9%)	2 (3.3%)		
IA2	53 (50.5%)	21 (35%)		
IB	8 (7.6%)	5 (8.3%)		
IC	1 (1%)	1 (1.7%)		
IIA	2 (1.9%)	5 (8.3%)		
IIB	11 (10.5%)	2 (3.3%)		
IIC	14 (13.3%)	18 (30%)		
III	13 (12.4%)	6 (10%)		
ER score, median (IQR)	6 (5, 7)	6 (5.25, 7)	0.808	0.885
PR score, median (IQR)	6 (4, 7)	6 (2, 7)	0.405	0.550
CPS			0.049 *	0.290
<1%	11 (10.5%)	0 (0%)		
1% ≤ CPS < 5%	37 (35.2%)	17 (28.3%)		
5% ≤ CPS < 10%	19 (18.1%)	13 (21.7%)		
10% ≤ CPS < 50%	25 (23.8%)	17 (28.3%)		
≥50%	13 (12.4%)	13 (21.7%)		

Abbreviations: MMRd, mismatch repair deficient; IQR, interquartile range; BMI, body mass index; LVSI, lymphovascular space invasion; LNM, lymph node metastasis; ER, estrogen receptor; PR, progesterone receptor; CPS, combined positive score. *, <0.05.

**Table 3:** Clinicopathological and PD-L1 Profiles of MutLα-Deficient Tumors Stratified by MMR Loss Patterns.

Characteristics	MLH1 and PMS2	MLH1	PMS2
	(*n* = 75)	(*n* = 7)	(*n* = 23)
Age, mean ± sd	55.9 ± 8.2	55.7 ± 10.5	54.3 ± 9.1
BMI, median (IQR)	24.8 (23.1, 27.4)	29.3 (27.0, 30.5)	25.3 (24.0, 29.2)
Pathology			
endometriod	70 (94.6%)	6 (85.7%)	21 (91.3%)
mixed	3 (4.1%)	0 (0%)	1 (4.3%)
neuroendocrine	0 (0%)	1 (14.3%)	0 (0%)
carcinosarcoma	0 (0%)	0 (0%)	1 (4.3%)
mucinous	1 (1.4%)	0 (0%)	0 (0%)
Differentiation			
G1	49 (66.2%)	5 (71.4%)	14 (60.9%)
G2	10 (13.5%)	1 (14.3%)	5 (21.7%)
G3	15 (20.3%)	1 (14.3%)	4 (17.4%)
Invasive			
absent	58 (78.4%)	6 (85.7%)	18 (78.3%)
present	16 (21.6%)	1 (14.3%)	5 (21.7%)
Myometrial invasion			
absent	2 (2.7%)	0 (0%)	0 (0%)
<1/2	54 (73%)	6 (85.7%)	18 (78.3%)
≥1/2	18 (24.3%)	1 (14.3%)	5 (21.7%)
LVSI			
absent	41 (55.4%)	4 (57.1%)	14 (60.9%)
local	18 (24.3%)	0 (0%)	9 (39.1%)
extensive	15 (20.3%)	3 (42.9%)	0 (0%)
Pelvic LNM			
absent	68 (90.7%)	5 (71.4%)	21 (91.3%)
present	7 (9.3%)	2 (28.6%)	2 (8.7%)
Aortic LNM			
absent	73 (97.3%)	6 (85.7%)	22 (95.7%)
present	2 (2.7%)	1 (14.3%)	1 (4.3%)
Cervical invasion			
absent	65 (87.8%)	7 (100%)	19 (82.6%)
present	9 (12.2%)	0 (0%)	4 (17.4%)
Stage 2009			
IA	48 (64.9%)	5 (71.4%)	16 (69.6%)
IB	13 (17.6%)	0 (0%)	3 (13%)
II	5 (6.8%)	0 (0%)	1 (4.3%)
IIIA	1 (1.4%)	0 (0%)	1 (4.3%)
IIIC1	5 (6.8%)	1 (14.3%)	1 (4.3%)
IIIC2	2 (2.7%)	1 (14.3%)	1 (4.3%)
Stage 2023			
IA1	3 (4%)	0 (0%)	0 (0%)
IA2	35 (46.7%)	4 (57.1%)	14 (60.9%)
IB	8 (10.7%)	0 (0%)	0 (0%)
IC	1 (1.3%)	0 (0%)	0 (0%)
IIA	1 (1.3%)	0 (0%)	1 (4.3%)
IIB	8 (10.7%)	1 (14.3%)	2 (8.7%)
IIC	11 (14.9%)	0 (0%)	3 (13%)
III	8 (10.7%)	2 (28.6%)	3 (13%)
ER score, median (IQR)	6 (4.5, 7)	5.5 (5, 6.125)	6.5 (6, 7.75)
PR score, median (IQR)	6 (4, 7)	4.5 (3.5, 5.25)	6.5 (6, 7)
CPS			
<1%	8 (10.7%)	1 (14.3%)	2 (8.7%)
1% ≤ CPS < 5%	28 (37.3%)	4 (57.1%)	5 (21.7%)
5% ≤ CPS < 10%	12 (16%)	2 (28.6%)	5 (21.7%)
10% ≤ CPS < 50%	14 (18.7%)	0 (0%)	11 (47.8%)
≥50%	13 (17.3%)	0 (0%)	0 (0%)

Abbreviations: IQR, interquartile range; BMI, body mass index; LVSI, lymphovascular space invasion; LNM, lymph node metastasis; ER, estrogen receptor; PR, progesterone receptor; CPS, combined positive score.

**Table 4:** Clinicopathological and PD-L1 Profiles of MutSα-Deficient Tumors Stratified by MMR Loss Patterns.

Characteristics	MSH2 and MSH6	MSH2	MSH6
	(*n* = 26)	(*n* = 3)	(*n* = 31)
Age, mean ± sd	52.4 ± 8.4	45 ± 6	58.7 ± 7.2
BMI, mean ± sd	25.1 ± 4.0	23.1 ± 2.0	25.0 ± 3.0
Pathology			
endometriod	21 (80.8%)	3 (100%)	25 (80.6%)
clear cell	2 (7.7%)	0 (0%)	3 (9.7%)
mixed	2 (7.7%)	0 (0%)	2 (6.5%)
gastrointestinal	1 (3.8%)	0 (0%)	0 (0%)
mesonephric	0 (0%)	0 (0%)	1 (3.2%)
Differentiation			
G1	13 (50%)	1 (33.3%)	19 (61.3%)
G2	2 (7.7%)	1 (33.3%)	3 (9.7%)
G3	11 (42.3%)	1 (33.3%)	9 (29%)
Invasive			
absent	17 (65.4%)	2 (66.7%)	22 (71%)
present	9 (34.6%)	1 (33.3%)	9 (29%)
Myometrial invasion			
absent	2 (7.7%)	1 (33.3%)	0 (0%)
<1/2	15 (57.7%)	1 (33.3%)	23 (74.2%)
≥1/2	9 (34.6%)	1 (33.3%)	8 (25.8%)
LVSI			
absent	18 (69.2%)	2 (66.7%)	17 (54.8%)
local	7 (26.9%)	0 (0%)	11 (35.5%)
extensive	1 (3.8%)	1 (33.3%)	3 (9.7%)
Pelvic LNM			
absent	24 (92.3%)	2 (66.7%)	28 (90.3%)
present	2 (7.7%)	1 (33.3%)	3 (9.7%)
Aortic LNM			
absent	26 (100%)	2 (66.7%)	30 (96.8%)
present	0 (0%)	1 (33.3%)	1 (3.2%)
Cervical invasion			
absent	22 (84.6%)	2 (66.7%)	26 (83.9%)
present	4 (15.4%)	1 (33.3%)	5 (16.1%)
Stage 2009			
IA	14 (53.8%)	1 (33.3%)	20 (64.5%)
IB	6 (23.1%)	0 (0%)	5 (16.1%)
II	4 (15.4%)	1 (33.3%)	3 (9.7%)
IIIC1	2 (7.7%)	0 (0%)	2 (6.5%)
IIIC2	0 (0%)	1 (33.3%)	1 (3.2%)
Stage 2023			
IA1	1 (3.8%)	1 (33.3%)	0 (0%)
IA2	8 (30.8%)	0 (0%)	13 (41.9%)
IB	2 (7.7%)	0 (0%)	3 (9.7%)
IC	1 (3.8%)	0 (0%)	0 (0%)
IIA	2 (7.7%)	1 (33.3%)	2 (6.5%)
IIB	0 (0%)	0 (0%)	2 (6.5%)
IIC	10 (38.5%)	0 (0%)	8 (25.8%)
III	2 (7.7%)	1 (33.3%)	3 (9.7%)
ER score, mean ± sd	5.2708 ± 2.8513	5.6667 ± 2.3094	5.7069 ± 2.2461
PR score, mean ± sd	4.375 ± 3.0512	3.8333 ± 3.3292	5.0714 ± 2.8045
CPS			
1% ≤ CPS < 5%	9 (34.6%)	1 (33.3%)	7 (22.6%)
5% ≤ CPS < 10%	3 (11.5%)	1 (33.3%)	9 (29%)
10% ≤ CPS < 50%	8 (30.8%)	0 (0%)	9 (29%)
≥50%	6 (23.1%)	1 (33.3%)	6 (19.4%)

Abbreviations: BMI, body mass index; LVSI, lymphovascular space invasion; LNM, lymph node metastasis; ER, estrogen receptor; PR, progesterone receptor; CPS, combined positive score.

## Data Availability

The data analyzed in this study is available from the corresponding authors on reasonable request.

## Supplementary Materials

The following supporting information can be downloaded at: https://www.mdpi.com/article/10.3390/cancers18182946/s1.
