# Supplementary material for: Heterogeneity in Clinicopathological and PD-L1 Expression Profiles Across Mismatch Repair Protein Deficiency Patterns in Endometrial Carcinoma
Source: Cancers (Basel). 2026 Sep 11;18(18):2946. doi: 10.3390/cancers18182946 (PMC13604280; doi:10.3390/cancers18182946)
Supplement: Supplementary file 1 [file cancers-18-02946-s001.zip › Supplementary Table S1.pdf]

Characteristics	MutL α (n=105)	MutSα (n=60)	MutL α +MutSα (n=8)	pvalue
**Age, median (IQR)**	55 (51, 61)	55 (49, 62)	54 (53.5, 58)	0.930
**BMI, median (IQR)**	25.0 (23.4, 28.6)	24.3 (23.0, 27.3)	22.5 (21.9, 28.5)	0.290
**Pathology**				0.042*
endometriod	98(93.3%)	49 (81.7%)	7 (87.5%)	
clear cell	0 (0%)	5 (8.3%)	0 (0%)	
mixed	4 (3.8%)	4 (6.7%)	0 (0%)	
other	3 (2.9%)	2 (3.3%)	1 (12.5%)	
**Differentiation**				0.186
G1	69 (65.7%)	33 (55%)	6 (75%)	
G2	16 (15.2%)	6 (10%)	1 (12.5%)	
G3	20 (19%)	21 (35%)	1 (12.5%)	
**Invasive**				0.221
absent	82 (78.8%)	41 (68.3%)	7 (87.5%)	
present	22 (21.2%)	19 (31.7%)	1 (12.5%)	
**Myometrial invasion**				0.449
absent	3 (2.9%)	3 (5%)	1 (12.5%)	
＜1/2	78 (74.3%)	39 (65%)	6 (75%)	
≥1/2	24 (22.9%)	18 (30%)	1 (12.5%)	
**LVSI**				0.395
absent	60 (57.1%)	37 (61.7%)	6 (75%)	
local	27 (25.7%)	18 (30%)	2 (25%)	
extensive	18 (17.1%)	5 (8.3%)	0 (0%)	
**Pelvic LNM**				1
absent	94 (89.5%)	54 (90%)	8 (100%)	
present	11 (10.5%)	6 (10%)	0 (0%)	
**Aortic LNM**				0.849
absent	101 (96.2%)	58 (96.7%)	8 (100%)	
present	4 (3.8%)	2 (3.3%)	0 (0%)	
**Cervical invasion**				0.741
absent	92 (87.6%)	50 (83.3%)	7 (87.5%)	
present	13 (12.4%)	10 (16.7%)	1 (12.5%)	
**Stage 2009**				0.840
IA	70 (66.7%)	35 (58.3%)	6 (75%)	
IB	16 (15.2%)	11 (18.3%)	1 (12.5%)	
II	6 (5.7%)	8 (13.3%)	1 (12.5%)	
IIIA	2 (1.9%)	0 (0%)	0 (0%)	
IIIC1	7 (6.7%)	4 (6.7%)	0 (0%)	
IIIC2	4 (3.8%)	2 (3.3%)	0 (0%)	
**Stage 2023**				0.128
IA1	2 (1.9%)	2 (3.3%)	1 (12.5%)	
IA2	53 (51%)	21 (35%)	5 (62.5%)	
IB	8 (7.7%)	5 (8.3%)	0 (0%)	

IC	1 (1%)	1 (1.7%)	0 (0%)	
IIA	2 (1.9%)	5 (8.3%)	1 (12.5%)	
IIB	11 (10.6%)	2 (3.3%)	0 (0%)	
IIC	14 (13.5%)	18 (30%)	1 (12.5%)	
III	13 (12.5%)	6 (10%)	0 (0%)	
**ER score, median (IQR)**	6 (5, 7)	6 (5.25, 7)	7.5 (6.75, 7.75)	0.257
**PR score, median (IQR)**	6 (4, 7)	6 (2, 7)	7 (6, 7.75)	0.228
**CPS score**				0.013*
＜1%	11 (10.5%)	0 (0%)	0 (0%)	
1%≤CPS＜5%	37 (35.2%)	17 (28.3%)	0 (0%)	
5%≤CPS＜10%	19 (18.1%)	13 (21.7%)	4 (50%)	
10%≤CPS＜50%	25 (23.8%)	17 (28.3%)	4 (50%)	
≥50%	13 (12.4%)	13 (21.7%)	0 (0%)	

Abbreviations: MMRd, mismatch repair deficient; MMRp, mismatch repair proficient; IQR, interquartile range; BMI, body mass index; LVSI, lymphovascular space invasion; LNM, lymph node metastasis; FIGO, International Federation of Gynecology and Obstetrics; ER, estrogen receptor; PR, progesterone receptor; CPS, combined positive score.
