# Supplementary material for: Heterogeneity in Clinicopathological and PD-L1 Expression Profiles Across Mismatch Repair Protein Deficiency Patterns in Endometrial Carcinoma
Source: Cancers (Basel). 2026 Sep 11;18(18):2946. doi: 10.3390/cancers18182946 (PMC13604280; doi:10.3390/cancers18182946)
Supplement: Supplementary file 1 [file cancers-18-02946-s001.zip › Supplementary Table S2.pdf]

Table S2. CPS positivity rates at different cutoffs vary between MutLα -deficient and MutSα
-deficient MMR subtypes.

**CPS cutoff**	**MMR status**	**N (Negative/Positive)**	**Cramer's V**	**P value**	**FDR q-value**
1%	**MutLα** -deficiency	11 / 94	0.177	0.023	0.046
	**MutSα** -deficiency	0 / 60			
5%	**MutLα** -deficiency	48 / 57	0.171	0.028	0.046
	**MutSα** -deficiency	17 / 43			
